# Effective Discussions of Affective Cases: A Survey of Attenders of the Mood Disorders Grand Rounds

**DOI:** 10.1192/bjo.2025.10315

**Published:** 2025-06-20

**Authors:** Joseph Thorne, Tiago Costa, Michael Browning, Stuart Watson

**Affiliations:** 1 Regional Affective Disorders Service, Newcastle upon Tyne, United Kingdom; 2 Oxford University, Oxford, United Kingdom

## Abstract

**Aims:** Specialist mood disorder services in the UK are diverse in structure and spread over different clinical-academic centres in the UK. Relationships between these centres are strong but often based on academic projects, with limited opportunities for clinical case discussions. The NIHR Mental Health Translational Research Collaboration, together with the ASCEnD trial team, has set up an online monthly meeting of tertiary mood disorder services: the Mood Disorders Grand Rounds (MDGR). The aims are: 1) to bring together people with expertise and interest from different centres across the UK; 2) to discuss complex and difficult to treat (or interesting) cases; 3) to consider treatment options. The format includes a 20-minute anonymised case presentation by a specialist, covering clinical and thematic aspects, followed by a 40-minute panel discussion focusing on case management, related themes, and relevant research studies. The presentership rotates between centres around the country and encourages a multidisciplinary approach.

Following the first 12 months of MDGR, we distributed a survey to evaluate and develop the meetings.

**Methods:** An evaluation form was developed and sent to all registered attendees over the course of six months, on a rolling basis. Participants were asked to both rate the effectiveness of various aspects of the programme and to submit suggestions for improvement, including suggestions for future speakers. Questions included both Likert scored items and free text responses.

**Results:** We received 21 responses (12% of those registered). 75% of respondents had not been to a similar regular collaborative programme previously. 50% of respondents stated that the MDGR had directly influenced their clinical practice, examples being of “Using MAOIs in a case where I hadn’t considered it before” and “identification of a patient with likely autoimmune encephalitis”. The remaining 50% stated that whilst the programme was relevant it had not had a direct result on practice.

**Conclusion:** A high proportion of respondents reported their clinical practice had been directly influenced by attendance. This suggests the MDGR is fulfilling the stated aim of focusing on clinical discussions and is of value to attenders. The rate of response is low and could be biased to those who found it more useful.

